# “Students want HIV testing in schools” a formative evaluation of the acceptability of HIV testing and counselling at schools in Gauteng and North West provinces in South Africa

**DOI:** 10.1186/s12889-015-1746-x

**Published:** 2015-04-17

**Authors:** Sphiwe Madiba, Mathilda Mokgatle

**Affiliations:** Department of Environmental and Occupational Heath, School of Public Health, Sefako Makgatho Health Sciences University, Pretoria, South Africa; Department of Biostatistics, School of Public Health, Sefako Makgatho Health Sciences University, Pretoria, South Africa

**Keywords:** Uptake of HTC, Acceptability of HTC at school, Students, South Africa, Sexual behaviour, Disclosure, HIV status, Stigma and discrimination

## Abstract

**Background:**

The proposal by the South African Health Ministry to implement HIV testing and counselling (HTC) at schools in 2011 generated debates about the appropriateness of such testing. However, the debate has been between the Ministries of Education and Health, with little considerations of the students. The main aim of the study was to assess the students’ opinions and uptake of HIV testing and counselling in general, and the acceptability of the provision of HIV testing and counselling in schools. The study also determined the association between socio-demographic characteristics, sexual behaviour, and HIV testing behaviour of the students.

**Methods:**

A survey was conducted among grade 10–12 high school students in North West and Gauteng provinces, South Africa. Seventeen high schools (nine rural and eight urban) were randomly selected for the administration of a researcher-assisted, self-administered, semi-structured questionnaire.

**Results:**

A total of 2970 students aged 14–27 years participated in the study; 1632 (55%) were girls, 1810 (61%) ever had sex, and 1271 (49.8%) had more than one sex partner. The mean age of first sexual activity was 15.6. Half (n = 1494, 50.1%) had been tested for HIV. Having multiple sexual partners, age, and gender were significantly associated with increased odds of having had a HIV test. Fear, being un-informed about HTC, and low HIV risk perceptions were the reasons for not getting tested. The acceptability of HTC at school was high (n = 2282, 76.9%) and 2129 (71.8%) were willing to be tested at school. Appropriateness, privacy, and secrecy were the main arguments for and against HTC at school. One-third (n = 860, 29%) had intentions to disclose their HIV status to students versus 1258 (42.5%) for teachers. Stigma, discrimination and secrecy were the primary reasons students did not intend to disclose.

**Conclusions:**

A high acceptability of HTC and willingness to be tested at school suggest that HIV prevention programs tailored to youth have a high potential of success given the readiness of students to uptake HTC. Bringing HIV testing to the school setting will increase the uptake of HTC among youth and contribute towards efforts to scale up HTC in South Africa.

## Background

Models of HIV testing, including voluntary counselling and testing (VCT), provider-initiated testing and counselling (PITC), home-based HIV counselling and testing (HBCT) and HIV self-testing, have been proposed and implemented over time, with the aim of increasing the uptake of HIV testing. Even though VCT has been the dominant model globally [1,2], its utilization remains low [3]. The Joint United Nations Programme on HIV/AIDS (UNAIDS) and the World Health Organization (WHO) recommended the implementation of PITC as an effective public health intervention to increase access to HIV counselling and testing in countries with generalized HIV epidemics [3]. In early 2010, VCT in South Africa expanded to include PITC, to ensure that HIV testing and counselling (HTC) becomes the standard of care in all consultations with health providers [4].

In South Africa, the PITC campaign aimed to test 15 million people for HIV within a year. To reach all South Africans, the campaign targeted 12- to 60-year-olds to ensure that all South Africans know their HIV status. The challenge is that over the years, counselling and testing for HIV, has been widely advocated as a HIV prevention strategy among adults. To ensure that young people are reached, the National Department of Health (NDoH) proposed that HTC be implemented at high schools to ensure that HIV testing is available for students. The question that needed to be answered after the proposal of HTC at schools was whether it could work as well for students at school as it did for adults. However, the debate on the appropriateness of HTC at schools has been between the Ministries of Education and Health, with little considerations of the students.

Although there have been limited studies on the use of HIV testing services by youth at schools in South Africa and sub-Saharan Africa, the focus of most studies on VCT has been on university and college students ([5-8]). A study conducted in Tanzania found that attitudes and practices towards HIV testing among secondary school students were influenced by the availability and accessibility of VCT centres. Schools, as well as hospitals, were mentioned as one of the preferred places where young people would like VCT services to be provided [9]. More than two decades ago, there were already indications that school-based clinics made HTC more accessible to young people than other health settings in the United States [10]. A decade later, a VCT program using forum drama to increase HIV awareness and testing in high schools in Kwa-Zulu Natal, in South Africa, also reported positive responses of students towards HIV testing and counselling at school [11].

Currently, HTC services in most countries in sub-Saharan Africa are offered in clinics, hospitals, through a door to door approach, and outreach campaigns, but there are no school based HTC services as yet. However, a mission hospital in Uganda was successful in providing mobile VCT services in school settings [12], this is the only documented model for providing HTC at schools. There has not been any empirical data on the benefits or harms of school based HIV testing in South Africa and other sub Saharan countries to inform the decisions of the Ministries of Education and Health. The uptake of the HTC program at schools will depend on the students’ willingness to utilize the service, and it was for this reason that we deemed them as appropriate to inform the implementation of the proposed HTC at school.

The main aim of our study was to assess the students’ opinions of, and uptake of HIV testing and counselling in general, and the acceptability of the provision of HIV testing and counselling in schools. The study also determined the association between socio-demographic characteristics, sexual behaviour, and HIV testing behaviour of the students. Understanding acceptability of HIV testing at schools from the perspective of students is essential to the success of the proposed roll out of the HTC campaign in schools. While an understanding of HTC practices and sexual behaviour will inform the design of appropriate interventions in order to increase uptake of HTC services amongst youth in school [13]. Improving access to appropriate HIV testing and counselling for young people is important for prevention of HIV transmission [14]. Therefore, schools have the potential to make valuable contributions to HIV prevention and other health programs because they harbour large numbers of youths.

## Methods

A formative evaluation design was employed to assess whether schools can be potential settings where HTC can be implemented. A survey was conducted among grade 10–12 high school students. The study settings consisted of 17 public high schools in the Bojanala educational district in North West province and the Tshwane North educational district in Gauteng province. The schools were randomly selected from 56 high schools, and all were eligible for inclusion in the study because they fall under the provincial departments of education and follow the same curriculum and enrolment processes. Of the 17 high schools included in the sample, nine were randomly selected from two rural sub-districts in Bojanala and eight from an urban district in Tshwane North.

Data collection occurred from July to September 2013, after permission to conduct the study was obtained from the relevant Departments of Education. Permission was also obtained from sub-district area managers who assisted the investigators in the random selection of the schools; they also facilitated access to the selected schools by informing the principals about the study. The individual principals then nominated a school contact, who was generally the Life Orientation (LO) teacher, to facilitate the actual administration of the survey. The research team then visited the selected schools to meet with the school contact to explain the purpose of the study and to discuss the protocol to be followed during the administration of the questionnaire. In all the schools surveyed, the LO periods on the school time table for grade 10–12 were randomly assigned to the research team on the day of data collection, and all students in the assigned LO periods volunteered to participate. The LO periods were offered for the administration of the questionnaire because the teachers were of the opinion that the purpose of the study and the questions that the students had to respond to were relevant content for the LO lessons. In 10 schools, only the LO periods in grades 10–11 were selected; grade 12 students were excluded because they were preparing for final-year exams. Prior to the completion of the questionnaire, the purpose of study was thoroughly explained to the students by the field workers, and the students were also informed that participation was voluntary and that their responses were confidential. Their anonymity was ensured by not gathering any personal information. Students were also informed that they could withdraw from the study, without any consequences, at any time should they wish to do so.

### Measures

The study instrument was a researcher-assisted, self-administered, semi-structured questionnaire that included the socio-demographic background of the students, their sexual behaviour, HTC practices, and the acceptability of HTC at school. To assess student’s sexual experiences, we asked if they ever had sex, their age at first sexual intercourse, if they currently had a sexual partner, and the number of sexual partners in the past year. Four main questions were asked to assess the opinions and usage of HTC; students were asked if they ever tested for HIV in the past year, the place where HIV test was obtained, reasons for not having been tested, and whether students should be tested for HIV. Two main questions were asked to assess the acceptability of HTC at schools; students were asked whether HTC at school is a good idea and their willingness to uptake HTC at school. The survey used a validated questionnaire from the Australian Secondary Students and Sexual Health Survey [15]. The HIV knowledge related questions, sexual behaviour questions, and HIV counselling and testing questions were not modified but were asked as they appear in the original questionnaire. We only added questions related to HIV testing and counselling at school. However, the questionnaire was pretested among 150 students in a high school that was not included in the survey, to assess whether the questions were comprehensible to students. We also assessed the clarity of instructions and estimated the time needed to complete the questionnaire. Based on the evaluation of the pre-test, we did not modify any of the questions. The South African Department of Health’s Ethics in Health Research guidelines state that adolescents over the age of 12 years are able to consent unassisted to research so long as it poses minimal risk and is unlikely to be objectionable to parents and community members [16]. Permission to conduct the study was granted by the relevant Departments of Education, sub-district area managers, and school principals who located the study within the Life Orientation curriculum. Therefore, students signed an informed consent or an assent form before completing the questionnaire. The questionnaire was in English and took about 20 minutes to complete. The research team received the completed questionnaires from the students and checked that all the fields in the questionnaire were completed, hence, no data were missing. Students were however informed that they have the right not to answer any question that they were not comfortable to answer. The research team consisted of a research coordinator and five trained fieldworkers.

### Ethics

The study was conducted after obtaining ethical approval from the Research and Ethics Committee of the University of Limpopo, Medunsa Campus (MREC/H/215/2012: PG).

### Data analysis

Descriptive statistics in the form of the frequencies and proportions for categorical variables were computed. Chi-squared tests were used to determine the association of categorical variables and gender. Univariate and multiple logistic regressions were performed to assess the association between the outcome variable (ever been tested for HIV) and independent variables, which were the socio-demographic and sexual behaviour variables. Univariate logistic regressions were also performed to establish the relationship between the outcome variable (acceptability of HTC at school) and the independent variables, which were gender and ever been tested for HIV. The adjusted and unadjusted odds ratios (OR) were calculated and confidence intervals (CI) were set at 95%; P-values of <0.05 were considered to be statistically significant. Data were analysed using STATA version 13.

## Results

### Description of study participants

Table 1 illustrates the demographics and sexual behaviors of the students as stratified by gender. A total of 2,970 students in grades 10–12 participated, of which over half (n = 1632, 55%) were girls, and the majority (n = 1345, 45%) were in grade 11. The students were aged between 14 and 27 years (M = 17.4, SD = 1.39); the majority (n = 2741, 92.3%) were aged between 14–19 years. Two-thirds (n = 1810, 61%) had sex, and the mean age of first sexual activity was 15.6 years. With regards to the number of sexual partners, 1278 (50.1%) had one sexual partner, 600 (23.5%) had two partners, while 671 (26.3%) had more than two sexual partners in the past year.

**Table 1:** **Demographic and sexual behaviour characteristics of grade 10–12 students segregated by gender**

**Variables**	**Total n = 2970**	**Female n = 1632**	**Male n = 1338**	**P value**
Age groups				0.000
14*–19 years	2741(92.3)	1535 (94.1)	1206 (90.1)	
20 years and more	229 (7.7)	97 (5.9)	132 (9.9)	
Mean age 17.4 years				
Grade				0.012
Grade 10	1223 (41.2)	637 (39.0)	586 (43.8)	
Grade 11	1345 (45.3)	754 (46.2)	591 (44.2)	
Grade 12	402 (13.5)	241 (14.8)	161 (12.0)	
Have boyfriend or girlfriend				0.424
No	600 (20.2)	321 (19.7)	279 (20.85)	
Yes	2370 (79.8)	1311 (80.3)	1059 (79.2)	
Ever had sex				0.000
No	1157 (39.0)	753 (46.2)	404 (30.2)	
Yes	1810 (61.0)	878 (53.8)	932 (69.8)	
Number of sexual partners in the past year				0.000
One partner	1278 (50.2)	878 (62.1)	400 (35.1)	
Two partners	600 (23.5)	317 (22.4)	286 (25.1)	
More than two partners	(26.3)	219 (15.5)	452 (39. 8)	

*Only one student was 14 years old.

### Uptake of HIV testing and counselling

Almost all (n = 2663, 89.7%) were of the opinion that students should be tested for HIV in general. In addition, the students were asked two questions to assess their uptake of HIV testing and counselling. They were asked a single question regarding whether they have ever been tested for HIV, with yes or no response, and for those who were tested, they were asked about the place the HIV testing took place. Half (n = 1494, 50.3%) of the students had been tested for HIV in the past year, 1090 (73.2%) used the HTC services in hospitals, 205 (13.8%) used clinics, and 193 (13%) used private medical practitioners. Almost an equal proportion (n = 1476, 49.7%) had not been tested, and they subsequently responded to an open-ended question and provided the reasons for not getting tested. The responses were categorised into themes, and the main reasons for not being tested were: fear of positive results 227 (17.2%), having no need for testing 218 (16.5%), being sexually inexperienced 178 (13.5%), not sure where to go for HIV testing 157 (11.9%), and being convinced that they were HIV negative 195 (14.8%).

Table 2 shows the univariate logistic regression of factors associated with having been tested for HIV in the preceding year. The results show that students who reported having a sexual partner were almost two times more likely (OR = 1.65, p = 0.000, CI: 1.38–1.98) to have had a HIV test than those who did not have a sexual partner. Sexually active students were two times more likely (OR = 2.25, p = 0.000, CI: 1.94–2.62) to have had a HIV test than those who were not sexually active. We also found that age and grade were also associated with the odds of having had a HIV test. Students in the older age group were two times more likely (OR = 2.24, p = 0.000, CI: 1.68–2.98) to have had a HIV test than those in the younger age group, and grade 11 and 12 students were more likely (1.21, p = 0.012, CI: 1.04-1.42, OR = 1.30, p = 0.023, CI: 1.03–1.62) to have had a HIV test than those in grade 10. The results further showed no association between HIV testing, number of sexual partners, and gender.

**Table 2:** **Demographic and sexual behaviour variables associated with testing for HIV in the previous year**

	**Not tested n = 1476**	**Tested n = 1494**	**Unadjusted OR (95% CI)**	**Adjusted OR (95% CI)**
Age groups				
14–19 years	1403 (51.1)	1338 (48.8)	Ref	
20 years and more	73 (31.9)	156 (68.1)	2.24 (1.68-2.98)	1.21 (1.13-1.30)*
Gender				
Female	819 (50.2)	813 (49.8)	Ref	
Male	657 (49.1)	681 (50.9)	1.04 (0.90-1.20)	0.92 (0.77-1.09)
Grades				
Grade 10	646 (52.8)	577 (47.1)	Ref	
Grade 11	644 (47.9)	701 (52.1)	1.21 (1.04-1.42)	
Grade12	186 (46.3)	216 (53.7)	1.30 (1.03–1.62)	
Have boyfriend or girlfriend				
No	358 (59.7)	242 (40.3)	Ref	
Yes	1118 (47.2)	1252 (52.8)	1.65 (1.38-1.98)	1.30 (0.98-1.73)
Ever had sex				
No	717 (62.0)	440 (38.0)	Ref	
Yes	759 (41.9)	1051(58.1)	2.25 (1.94-2.62)	1.97 (1.63-2.38)*
Number of sexual partners in the past year				
One partner	608 (49.5)	670 (50.6)	Ref	
More than one partners	621 (50.5)	653 (49.4)	0.95 (0.81-1.11)	0.84 (0.71-0.99)*

OR: unadjusted/unadjusted odds ratio; CI: 95% confidence interval; *significant p value.

The result of the multivariate analyses showed that, age (OR = 1.70, p = 0.002, CI: 1.21-2.38), ever had sex (OR = 1.97, p = 0.000, CI: 1.63-2.38), and number of sexual partners (OR = 0.84, p = 0.046 CI: 0.71-0.99) were significantly associated with the odds of testing for HIV.

### Acceptability of HTC at school

Acceptability of HTC at school was assessed using a single question: Is having HIV testing and counselling at school a good idea? The possible responses were: agree, disagree or not sure. The results showed that HTC at school was highly acceptable, more than three-quarters (n = 2282, 76.9%) of the students were in agreement with the statement that having HIV testing and counselling at school is a good idea (Table 3). A single question with yes or no responses was used to assess students’ willingness to take up HTC if it was offered at school, and three-quarters (n = 2129, 71.8%) were willing to undergo HTC at school. For those who were not willing to be tested at school, they were asked for the reasons for the unacceptability of testing at school using an open-ended question. The responses were categorised into themes, and the main reasons were: being scared to be tested at school (n = 80, 23.5%), that school is not the right place for HIV testing (n = 49, 14.4%), unwillingness to be tested (n = 33, 9.7%), fear of gossip (n = 33, 9.7%) and lack of privacy (n = 31, 9.1%).

**Table 3:** **Grade 10–12 students’ responses and opinions on the acceptability of HIV testing and counselling at school**

**Questions**	**Response**	**Frequency/Percentage**
Students should be tested for HIV?	Yes	2663 (89.7)
	No	102 (3.4)
	Not sure	204 (6.9)
Having HIV counselling and testing in school is a good idea	Agree	2282 (76.9)
	Disagree	267 (9.0)
	Not sure	419 (14.1)
Would you be willing to take up HTC if it was offered in school?	Yes	2129 (71.8)
	No	368 (12.4)
	Not sure	469 (15.8)
If you test positive would you disclose your status to other students?	Yes	860 (29.0)
	No	1031 (34.8)
	Not sure	1074 (36.2)
If you test positive would you disclose your status to the teachers?	Yes	1258 (42.5)
	No	766 (25.9)
	Not sure	937 (31.6)

Concerning students’ intentions to disclose their HIV status to other students and teachers if tested at school, only one-third (n = 860, 29%) would tell other students, while 1258 (42%) would tell their teachers (Table 3).

An open-ended question was posed to those who had no intention of disclosing their HIV status to other students and teachers, and the most cited reasons why students would not disclose their status included: HIV status is a personal secret 274 (34.8%), being scared to tell other students 151 (19.2%), fear of being judged and discriminated against 118 (15%), fear of being gossiped about 92 (11.7%), fear of being isolated and treated badly 73 (9.3%), and fear of being belittled 57 (7.2%). Students cited similar reasons for not disclosing to teachers, but also mentioned that teachers cannot keep a secret.

### Relationship of HTC to gender, being tested and disclosure

The results showed that among the students who reported being tested for HIV (n = 1474) and 50.2% were girls. A higher proportion of girls as compared with boys (92% vs. 86.9%) were also of the opinion that students should be tested, and this difference was statistically significant (OR = 0.57, p = 0.000, CI: 0.45-0.73). The results further showed that more girls compared with boys (78% vs. 75%) thought that having HTC at school was a good idea and 76% of girls versus 67% of boys were willing to use HTC services at school if it was available. Logistic regression showed that girls were more positively associated with a willingness to be tested at school (OR = 0.65, p = 0.000, CI: 0.55–0.76), although there was no significant difference regarding the acceptability of HTC at school between boys and girls. With regards to the disclosure of HIV positive results to other students and teachers, more boys compared with girls (31% vs. 27%) said they would disclose their test results to other students and these differences were statistically significant (OR = 1.19, p = 0.000, CI: 1.01-1.40), and 44% of boys vs. 41% of girls would disclose their HIV status to teachers and these differences were not statistically significant (Table 4).

**Table 4:** **Acceptability of HIV testing and counselling at school by gender**

**Variables**	**Female**	**Male**	**Total**	**OR (Con. Interval)**
Tested for HIV				
No	819 (50.2)	657 (49.1)	1476 (49.7)	Ref
Yes	813 (49.8)	681 (50.9)	1494 (50.3)	1.04 (0.90-1.20)
Students should be tested				
No	131 (8.0)	175 (13.1)	306 (10.3)	Ref
Yes	1501(92.0)	1162 (86.9)	2663 (89.7)	*0.57 (0.45-0.73)
HTC at school is a good idea				
Disagree	355 (21.8)	331 (24.8)	686 (23.1)	Ref
Agree	1276 (78.2)	1006 (75.2)	2282 (76.9)	0.84 (0.71-1.00)
Willing to be tested at school				
No	396 (24.3)	441 (33.0)	837 (28.2)	Ref
Yes	1233 (75.7)	896 (67.0)	2129 (71.8)	*0.65 (0.55-0.76)
Disclose to other students				
No	1185 (72.7)	920 (69.0)	2105 (71.0)	Ref
Yes	446 (27.3)	414 (31.0)	860 (29.0)	*1.19 (1.01-1.40)
Disclose to teachers				
No	958 (58.8)	745 (56.0)	1703 (57.5)	Ref
Yes	672 (41.2)	586 (44.0)	1258 (42.5)	1.12 (0.96-1.29)

OR: unadjusted odds ratio; CI: 95% confidence interval; *significant p value.

### Being tested and the acceptability of HTC

Half of the students had been tested for HIV, and the results showed that a higher proportion of students who had been tested for HIV were of the opinion that students should be tested than those who had not been tested (93.4% vs. 86%). We found a significant association between ever tested and opinions that learners should be tested (OR = 2.30, p = 0.000, CI: 1.78–2.95), willingness to be tested at school (OR = 1.37, p = 0.000, 1.17-1.61), disclosure of HIV status to students (OR = 1.32, p = 0.001, CI: 1.13-1.55), and disclosure to teachers (OR = 1.25, p = 0.002, CI: 1.08-1.45). There were no significant differences regarding the acceptability of HTC at school among students who were tested and those who were never tested (Table 5).

**Table 5:** **The acceptability of the implementation of HIV testing and counselling at school among ever tested and never tested students**

**Variables**	**Not tested n = 1476**	**Tested n = 1494**	**Total n = 2970**	**OR (Con. Interval)**
Students should be tested				
No	207 (14.0)	99 (6.6)	306 (10.3)	Ref
Yes	1268 (86.0)	1395 (93.4)	2663 (89.7)	*2.30 (1.78-2.95)
HTC at school is a good idea				
Disagree	357 (24.2)	329 (22.0)	686 (23.1)	Ref
Agree	1117 (75.8)	1165 (78.0)	2282 (76.9)	1.13 (0.95-1.34)
Willing to be tested at school				
No	464 (31.5)	373 (25.0)	837 (28.2)	Ref
Yes	1010 (68.5)	1119 (75.0)	2129 (71.8)	*1.37 (1.17-1.61)
Disclose to other students				
No	1088 (73.9)	1017 (68.1)	2105 (71.0)	Ref
Yes	384 (26.1)	476 (31.9)	860 (29.0)	*1.32 (1.13-1.55)
Disclose to teachers				
No	887 (60.3)	1017 (68.1)	1703 (57.5)	Ref
Yes	583 (39.7)	675 (45.3)	1258 (42.5)	*1.25 (1.08-1.45)

OR: unadjusted odds ratio; CI: 95% confidence interval; *significant p value.

## Discussion

This study assessed the acceptability of the provision of HTC at schools among high school students in rural and urban districts in South Africa. Students’ uptake of HTC showed that half had been tested for HIV in the previous year, which was low given that two-thirds of the students had engaged in sex and half had more than one sexual partner in the past year. Having multiple sexual partners, age, and gender were associated with increased odds of having had a HIV test. In the current study and others, young people who reported no sexual activity had a low perception of personal risk to HIV infection, and thought that there was no need for HIV testing [5,8,17-19], because, commonly, HTC services are associated with certain health needs, such as access to ART [20]. It is important that youth be targeted for HTC awareness and promotion to normalize HIV testing among healthy young people. The LO program could be a crucial vehicle for health education and for promoting the benefits of HTC.

The low uptake of HTC, risky sexual behaviours, and low risk perception among students are major public health concerns and justify the South African Government’s proposed HTC campaign targeting children from 12 years of age. HTC has been found to be an effective means of reducing the HIV/AIDS burden, and it is an important component of HIV/AIDS prevention strategies. HTC interventions empower uninfected individuals to protect themselves from HIV infection, and assist infected persons in adopting a healthier sexual lifestyle [5,8,18].

Despite the low uptake of HTC among students prior to the study, almost all (90%) felt that students should be tested for HIV. Our data also indicate a high level of acceptability (76.9%) of the implementation of HTC services for students at schools, and only a small percentage (10%) of students were not willing to be tested. These findings have implications for HIV prevention programs in South Africa because the uptake of HTC is essential for the prevention and control of HIV among youth, a population at high risk of HIV infection. These findings further suggest that HIV prevention programs tailored to youth have a high potential of success given the readiness of students to uptake HTC.

The study also found that three-quarters of the students were willing to be tested at school should HTC be implemented. The high willingness to be tested at school is different from the actual practice of low HTC uptake among the students. One of the challenges for the uptake of HTC services in South Africa and other countries is that HTC services are predominantly hospital and clinic-based, resulting in students in school being disproportionately disadvantaged because they do not utilize clinic services as often as their adult counterparts [21]. The reasons for the high acceptability and willingness to be tested might be that students perceive HTC at school as an appropriate program that is intended solely for them. HTC at school also has the potential to offer the confidentiality of HIV test results and the privacy of testing, the lack of which are often linked with a low utilization of HTC services [5,22].

We found that the proportion (10%) of students who said that HTC at school is not acceptable, believed that school is not the right place to be tested for HIV. Their major concern was that other students might witness their reaction to positive test results, that they will be subjected to gossip, and that other students will judge them. They were not certain that there would be privacy during testing and that the test results would be kept secret, as they anticipated that large groups of students may be tested at the same time. These findings should be taken into consideration during the implementation of HTC at schools, as a lack of privacy and confidentiality might pose as major barriers to the uptake of HTC among students. The issuing of HIV test results is one of the key issues in the proposed rollout of HTC that should be further explored with HIV prevention program designers and students to ensure the protection of the HIV status of the students. The concerns surrounding the issuing of HIV positive test results have been documented among the adult population utilizing HTC services [23-26].

The successful implementation of HTC at schools will depend on students’ acceptability of the service, as well as other fundamental principles of HTC programs, such as the disclosure of HIV status. Our data indicated low intentions of disclosure to students and teachers. Students who did not intend to disclose to teachers were of the opinion that teachers do not necessarily keep secrets. Keeping their HIV positive status secret is crucial for students because the provision of confidentiality and privacy of HIV test results was one of the conditions for them to be tested at school. It is important that the rollout of the proposed HTC at schools prepares teachers for their roles in supporting students to uptake HTC and to manage their HIV status.

Although the word stigma was not mentioned by the students as part of their fear of disclosure; isolation, humiliation, belittling, gossip and being judged are all acts of stigmatization [27]. These results reveal that stigma and discrimination are the primary reasons students did not intend to disclose their HIV status.

Our study findings should be interpreted in light of some limitations. We only included high schools in two sub-districts from two provinces; thus, the study does not provide a national picture of HTC acceptability. One of the limitations is that the study is based on self-reports by students who might have provided socially desirable responses regarding the issue of sexuality, which might have resulted in over- and under-reporting. However, the anonymity of the questionnaire might have encouraged students to be honest in answering the questions. The strength of the study is that it was conducted among students in the school setting where the programme will be implemented. The findings give an indication of the potential for programme uptake and utilization. Further studies are needed to determine whether our findings are representative of the situation in other provinces in South Africa.

## Conclusions

The study showed that HTC uptake was low. Fear, being un-informed about HTC services, and low personal risk perceptions of HIV infection were the main reasons for not being tested. The LO program should incorporate HIV testing and counselling in the curriculum to create awareness on the benefits of HTC and increase its uptake among students.

Our study findings also demonstrated that the acceptability of HTC at school is high, and there was willingness to uptake the service among students, even though only 50% of the students have ever tested. We found that HTC at school was highly acceptable because students perceived it as appropriate, convenient, and having the potential to offer the confidentiality of HIV test results and private testing.

Bringing HIV testing to the school setting will address some of the commonly cited barriers to HIV testing such as distance to HIV testing sites, lack of confidentiality of HIV test results, and privacy of testing, which often results in stigmatization. The results have shown that HIV prevention programs that are appropriate and intended for young people have the potential for high intake. We found that three quarters of the students were willing to test at school should HCT be implemented. Bringing HIV testing to the school setting will increase the uptake of HTC among youth and contribute towards efforts to scale up HTC in South Africa, but program designers should take into considerations the views and perspectives of students particularly on issues of confidentiality of test results. We found that similar to the adult population, fear of being stigmatized remains one of the challenges for testing and disclosure of HIV status among students.

In planning to roll out HTC at schools, there is also a need for interventions and educational programs to create and increase awareness to reduce stigmatisation and discrimination to assist students to accept living with HIV and also to be tolerant of HIV-positive persons. The packaging of the HTC programme should consider incorporating interventions to reduce risky sexual behaviour. The interventions should address sexual education, provide reproductive health information, prevent teenage pregnancy, and promote disclosure to sexual partners to prevent HIV transmission, assist students in delaying their sexual debut, promote secondary abstinence, and increase self-efficacy to negotiate consistent condom use.
